# SARS-CoV-2 E protein interacts with BRD2 and BRD4 SEED domains and alters transcription in a different way than BET inhibition

**DOI:** 10.1007/s00018-024-05343-8

**Published:** 2024-07-27

**Authors:** Nieves Lara-Ureña, Elena Gómez-Marín, Isabel Pozuelo-Sánchez, José C. Reyes, Mario García-Domínguez

**Affiliations:** 1 Department of Cell Dynamics and Signaling, Andalusian Centre for Molecular Biology and Regenerative Medicine-CABIMER, CSIC-Universidad de Sevilla-Universidad Pablo de Olavide, Av. Américo Vespucio 24, Seville, 41092 Spain; 2 Department of Genome Biology, Andalusian Centre for Molecular Biology and Regenerative Medicine-CABIMER, CSIC-Universidad de Sevilla-Universidad Pablo de Olavide, Av. Américo Vespucio 24, Seville, 41092 Spain; 3 Córdoba, Spain

**Keywords:** COVID-19, Bromodomain, JQ1, Interferon response, PROTAC

## Abstract

Bromodomain and extra-terminal (BET) proteins are relevant chromatin adaptors involved in the transcriptional control of thousands of genes. Two tandem N-terminal bromodomains are essential for chromatin attachment through acetyl-histone recognition. Recently, the BET proteins members BRD2 and BRD4 were found to interact with the SARS-CoV-2 envelope (E) protein, raising the question of whether the interaction constitutes a virus hijacking mechanism for transcription alteration in the host cell. To shed light on this question, we have compared the transcriptome of cells overexpressing E with that of cells treated with the BET inhibitor JQ1. Notably, E overexpression leads to a strong upregulation of natural immunity- and interferon response-related genes. However, BET inhibition results in the downregulation of most of these genes, indicating that these two conditions, far from causing a significant overlap of the altered transcriptomes, course with quite different outputs. Concerning the interaction of E protein with BET members, and differing from previous reports indicating that it occurs through BET bromodomains, we find that it relies on SEED and SEED-like domains, BET regions rich in Ser, Asp, and Glu residues. By taking advantage of this specific interaction, we have been able to direct selective degradation of E protein through a PROTAC system involving a dTAG-SEED fusion, highlighting the possible therapeutic use of this peptide for targeted degradation of a viral essential protein.

**Supplementary Information:**

The online version contains supplementary material available at 10.1007/s00018-024-05343-8.

## Introduction

**Fig. 1:**
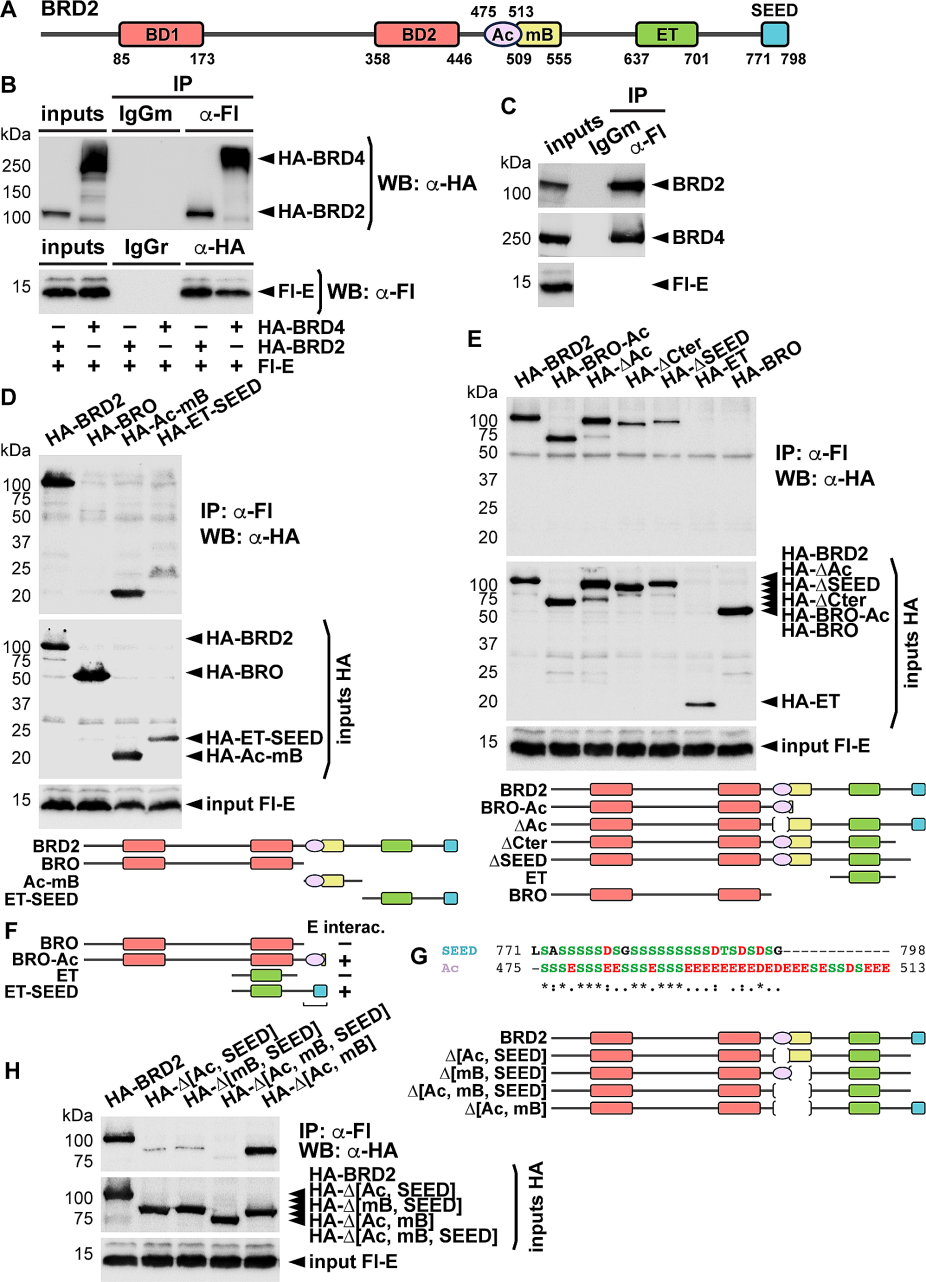
The SEED domain of BET proteins is involved in interaction with SARS-CoV-2 E protein. **A** Schematic representation of BRD2 domains. Numbers indicate amino acid positions. BD, bromodomain; Ac, acidic region; mB, motif B; ET, extra terminal domain; SEED, Ser, Asp, and Glu rich region. **B** HEK293T cells were transfected with expression constructs for Flag (Fl)-tagged E protein or HA-tagged BRD2 or BRD4 as indicated. Immunoprecipitation (IP) experiments were conducted with anti-Fl antibodies or normal mouse IgG as a control, followed by anti-HA western blot (WB), or with anti-HA antibodies or normal rabbit IgG as a control, followed by anti-Fl WB. **C** Extracts from cells transfected with Fl-E expression construct were immunoprecipitated with anti-Fl antibodies and analyzed by WB with antibodies against endogenous BRD2 or BRD4. Normal mouse IgG was used as a control. **D**, **E**, **H** The indicated HA-tagged constructs were transfected together with the Fl-E construct, and cells were processed for IP with anti-Fl antibodies followed by WB with anti-HA antibodies. A schematic representation of the different constructions in each set of analyses is included in each corresponding panel. BRO, N-terminal construction encompassing both bromodomains. **F** Summary of relevant results from (D) and (E). The small regions in BRO-Ac and in ET-SEED presumably mediating the interaction are underlined. **G** Alignment of SEED and Ac (SEED-like) of BRD2 by Clustal Omega. Numbers indicate amino acid positions. *, : and . denote fully conserved residues, conservation between groups of strongly similar properties, and conservation between groups of weakly similar properties, respectively. **B**-**E**, **H** 5% of each immunoprecipitated extract was loaded as input

Bromodomain and extra-terminal (BET) proteins are key transcriptional co-regulators involved in the control of many cellular processes [1]. The vertebrate family is formed by 4 members (BRD2, BRD3, BRD4 and BRDT). Excepting BRDT, whose expression is restricted to the male germ line, the other members are expressed ubiquitously. Structurally (Fig. 1A), they present two tandem bromodomains at the N-terminus for the recognition of acetyl groups, and an extra terminal (ET) domain at the C-terminus, specific to BET proteins and mediating protein-protein interactions. Between the bromodomains and the ET domain, a dimerization domain, the motif B (mB), which includes a coiled-coil structure, is also present [2]. Downstream to the ET domain, a region poorly characterized and rich in Ser, Asp, and Glu residues has been denominated SEED domain. BRD4 and BRDT additionally display a characteristic C-terminal domain (CTD) involved in contact with the RNA polymerase II [3, 4], while BRD2 displays an exclusive acidic region (Ac) before the mB, involved in specific protein interactions [5].

Altered expression of BET proteins is associated with relevant human diseases, especially cancer [6]. This explains why a great effort has been made to identify BET inhibitors, able to detach BET proteins from the chromatin, and BET degraders, to be used in therapies against cancer and different inflammatory diseases [7–9]. Among the major functions of BET proteins is the response to viral infection. The relationship of BET proteins with viruses is double. On one hand, BET proteins are key regulators of the immune response [10], but on the other hand, the interaction of a variety of viral proteins with different BET domains, to hijack BETs’ transcriptional function for its benefit, has been widely documented [11, 12].

Recently, a proteomic study on cell interactors of the different SARS-CoV-2 proteins revealed BRD2 and BRD4 interacting with the envelope (E) viral protein [13]. This has opened new hopes in fighting SARS-CoV-2 and the associated COVID-19 disease [14], since they are druggable proteins [9]. The E protein is a small polypeptide with relevant functions in virion maturation and assembly, which has been also suggested to play important roles in infection (reviewed in [15]). It is highly expressed but only a small fraction is directed to the viral membrane; so, most of the protein localizes at intracellular transport sites like the Golgi apparatus and the endoplasmic reticulum (ER) [15]. Structurally, the protein has a middle transmembrane domain, with an N-terminal region exposed to the cytoplasm and an intra-virion C-terminal domain [16]. In addition, oligomerization of E protein enables it to act as a viroporin for ion permeation [15].

Due to sequence similarity between histone H2A N-terminus and a region of E protein, it was indicated that E protein could interact with BETs through the bromodomains [13, 17, 18]. As they are essential for chromatin association and thereby for transcriptional activity, this raises the question of the possible interference of E protein with BET function and whether or not the interaction constitutes a SARS-CoV-2 hijacking mechanism in host cells. To address this subject, we have studied the transcriptional consequences of overexpressing the E protein to compare with the effects of BET inhibition. We found that these treatments cause quite different responses. Strikingly, concerning natural immunity and interferon response genes, they show opposite effects. We were unable to detect an interaction between E protein and BRD2 or BRD4 bromodomains. However, we uncovered an interaction between the intra-virion domain of E and the SEED domain of BET proteins and another E interactor. Furthermore, as a proof of concept, we have shown that a peptide including the SEED domain fused to the dTAG can direct E protein for proteasomal degradation, underlining the possible use of this molecule in therapies directed to promote selective degradation of a protein that is required for the virus life cycle.

## Results

### SARS-CoV-2 E protein interacts with SEED domains

SARS-CoV-2 E protein has been reported to interact with both BRD2 and BRD4 [13]. Thus, we first tested interaction in our selected model of study, HEK293T cells, widely used for SARS-CoV-2 studies [19, 20]. We expressed Flag-tagged E protein together with HA-tagged BRD2 or BRD4 and analyzed co-immunoprecipitation products using both anti-Flag and anti-HA antibodies. In both cases, the interaction was confirmed (Fig. 1B). To further check the interaction, we also checked that Flag-E could co-immunoprecipitate endogenous BRD2 and BRD4 (Fig. 1C). As in other cell systems it has been indicated that E-BET interaction occurs through the bromodomains [13, 17, 18], we tested this in our cells. We initially focused on BRD2 as it naturally lacks the exclusive CTD present in BRD4. Surprisingly, the N-terminal part of BRD2, encompassing both intact bromodomains, was unable to interact with E protein. However, the C-terminal part of BRD2 did interact with it (Fig. 1D). Even more, intriguingly, C-terminal BRD2 was split into two fragments for analysis, and each fragment was independently able to interact with E (Fig. 1D). One of the fragments corresponded to the mB together with the Ac region, and the other contained the rest of the C-terminus. We realized that these fragments have in common the presence of coiled-coil structures, one that we described inside the mB [2] and another described later upstream of the SEED region [21]. However, the independent or combined mutation of both coiled-coils did not affect the interaction (Supplementary Fig. S1). Thus, we decided to test additional BRD2 constructions for interaction (Fig. 1E). From analysis to this point, and as summarized in Fig. 1F, we observed that while the bromodomains were unable to interact with E protein, just addition of the Ac region allowed the interaction. Also, while the region containing the ET domain was unable to interact with E, just the addition of the SEED region facilitated the interaction. Therefore, we concluded that the SEED domain and the Ac were responsible for interacting with E-protein. Indeed, the Ac region is highly similar to the SEED region and constitutes a SEED-like domain (Fig. 1G). To confirm this, we analyzed constructions lacking these small domains. As observed in Fig. 1H, the combined deletion of both severely affected the interaction, but some products were still co-immunoprecipitated. We wondered if the presence of the mB could be interfering with the analysis since, as we have described, being the mB a dimerization motif, the endogenous protein with intact SEEDs can interact with E protein and dimerize with our SEED mutants, leading to indirect co-immunoprecipitation. We demonstrated that deleting both SEEDs, together with the mB, efficiently impaired the interaction with E (Fig. 1H). In the absence of mB, the lack of canonical SEED alone also affected the interaction significantly (Fig. 1H).

**Fig. 2:**
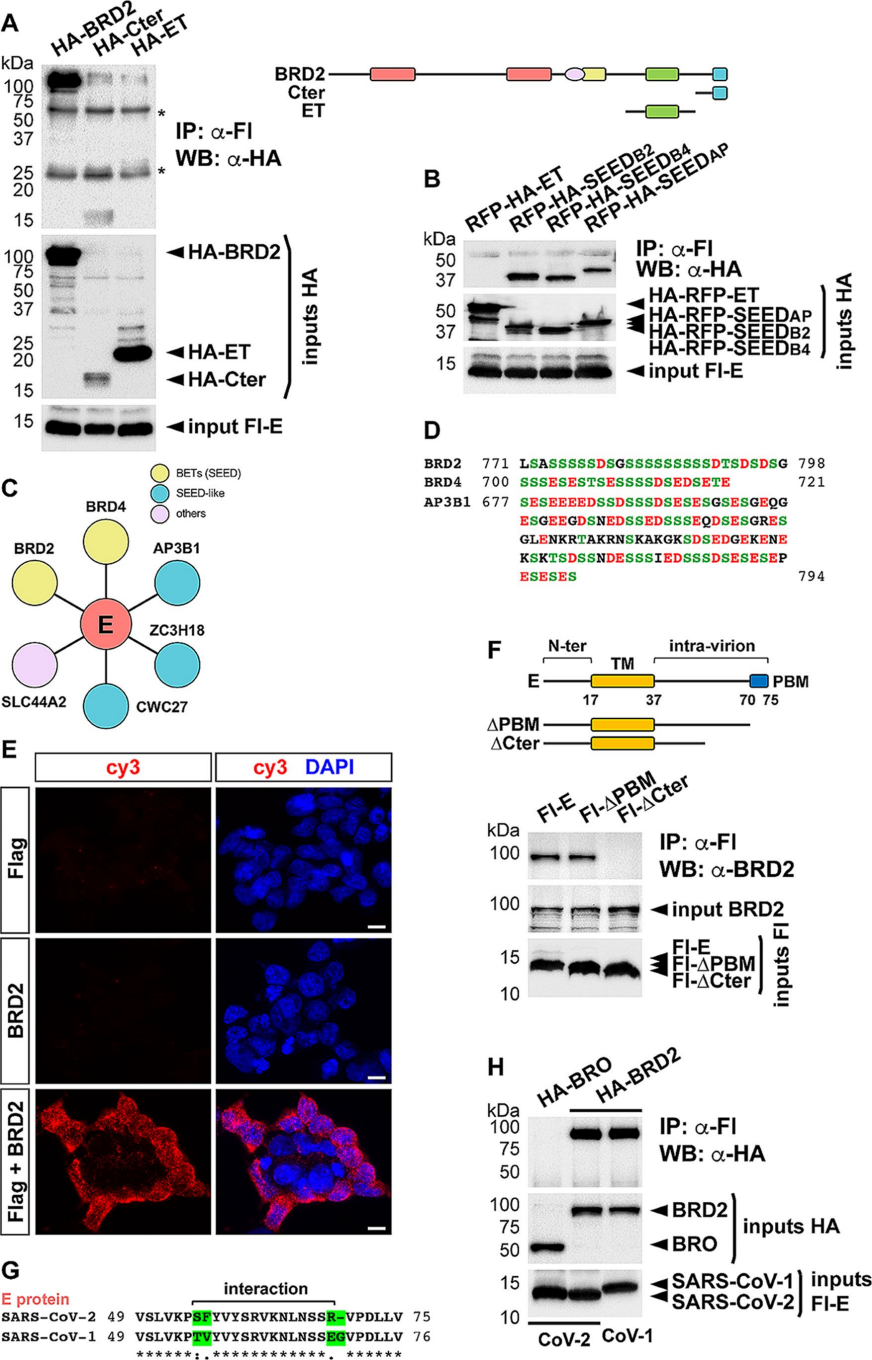
The SEED domains mediate interaction with the intra-virion region of E. **A** The indicated HA-tagged constructs were transfected together with the Flag (Fl)-E construct in HEK293T cells for immunoprecipitation (IP) experiments with anti-Fl antibodies followed by anti-HA western blot (WB). A schematic representation of the different constructions used is included in the panel. * IgG bands. **B** SEED domains from BRD2 (B2) and BRD4 (B4) and SEED-like domain from AP3B1 (AP), tagged with HA and fused to the Red Fluorescent Protein (RFP), were tested for IP with anti-Fl antibodies, followed by anti-HA WB, after expression in HEK293T cells together with Fl-E. RFP-HA-ET was used as a negative control. **C** Picture of E protein interactors described by Gordon et al. [13]. BET proteins are in yellow and other proteins with SEED-like domains are in blue. **D** Sequences of the SEED domains of BRD2 and BRD4, and the SEED-like domain of AP3B1. **E** In situ detection of the interaction between expressed Fl-E and endogenous BRD2 through a PLA assay. Antibodies against Flag and BRD2 were used together, or separately as negative controls. PLA signal is shown in red (cy3 fluorochrome) while DAPI-staining of nuclei is shown in blue. Scale bar 10 μm. **F** The indicated Fl-tagged E protein constructs were transfected in HEK293T cells for IP experiments with anti-Fl antibodies followed by detection of endogenous BRD2 by WB. A schematic representation of the different constructs used is included in the panel. TM, transmembrane domain; PBM, PDZ binding motif. **G** Fragments of the intra-virion region of SARS-CoV-1 and SARS-CoV-2 E proteins were aligned by Clustal Omega. *, : and . denote fully conserved residues, conservation between groups of strongly similar properties, and conservation between groups of weakly similar properties, respectively. **D**, **F** (upper part), **G** Numbers indicate amino acid positions. **H** The indicated Fl-tagged constructs involving SARS-CoV-2 and SARS-CoV-1 E proteins in combination with expression constructs for HA-BRO or HA-BRD2 as indicated, were transfected in HEK293T cells for IP experiments with anti-Fl antibodies followed by detection of HA-tagged proteins by WB. **A**, **B**, **F**, **H** 5% of each immunoprecipitated extract was loaded as input

To confirm that the SEED domain is sufficient for the interaction, a small construction with BRD2 C-terminus containing the SEED was tested for co-immunoprecipitation by Flag-E. This fragment was efficiently precipitated and not the ET domain used as a control (Fig. 2A). Even more, just the SEED, whether it is from BRD2 or BRD4, fused to the Red Fluorescent Protein (RFP) and tagged with HA was also precipitated by Flag-E, while an RFP-HA-ET protein was not precipitated (Fig. 2B). Interestingly, from the other four proteins reported to interact with E protein (Fig. 2C) [13] we identified that at least three of them contained SEED-like domains or acidic patches resembling SEED: AP3B1, ZC3H18 and CWC27. Thus, we also tested one of them, the SEED-like domain from AP3B1 (Fig. 2D), fused to RFP, for co-immunoprecipitation by Flag-E, which resulted in efficient precipitation as shown in Fig. 2B.

To evaluate the proximity of the interacting proteins we used a Proximity Ligation Assay (PLA) using primary antibodies coupled to specific secondary antibodies bound to unique DNA probes that can be amplified and detected by fluorescence when they are in close proximity (< 40 nm). The use of antibodies against endogenous BRD2 and Flag (for detection of expressed Flag-tagged E), revealed a strong PLA signal absent in negative controls, indicating that interacting proteins were in close proximity (Fig. 2E).

Finally, we also mapped the region of E involved in the interaction. To this purpose, we analyzed serial deletions from the E C-terminus, since the presence of a PDZ binding motif (PBM) in the C-terminus of E, just at the end of the intra-virion region, has been previously described [22, 23]. As shown in Fig. 2F, deletion of the PBM did not alter the interaction, but deletion of half the intra-virion region completely impaired it. This indicates that amino acids 55 to 69 are required for the interaction. Interestingly, this region contains the unique four residues not conserved between the original sequence reported for SARS-CoV-2 E protein and that from SAR-CoV-1 E (Fig. 2G). However, these differences did not prevent the interaction of SARS-CoV-1 E with BRD2, as shown in Fig. 2H.

### BET bromodomains are not required for interaction with the SARS-CoV-2 E protein

**Fig. 3:**
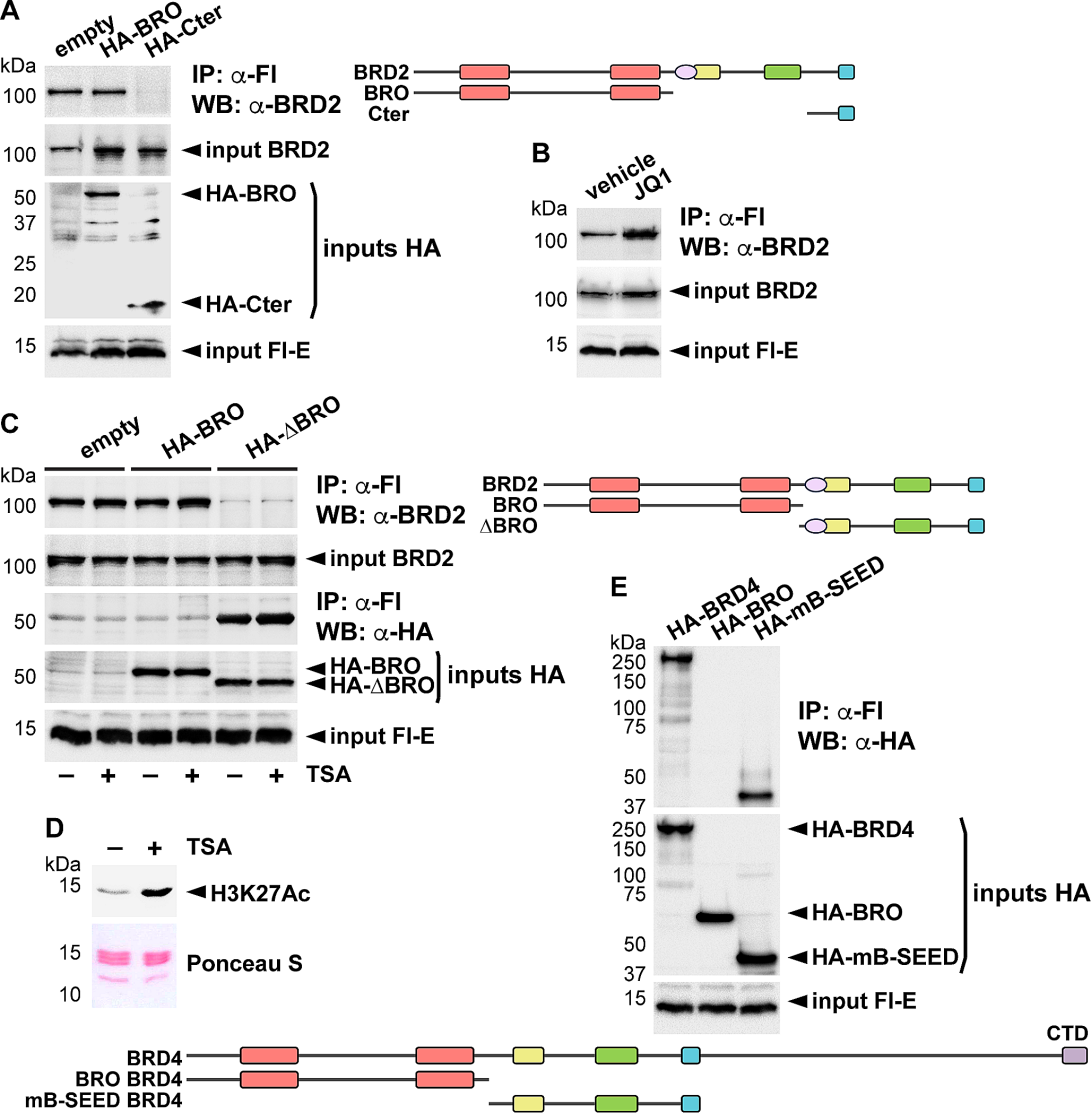
Bromodomains are not required for interaction with E protein. **A** The indicated HA-tagged constructs of BRD2 were expressed as indicated in HEK293T cells, together with Flag (Fl)-E to test how they compete anti-Fl-mediated immunoprecipitation (IP) of endogenous BRD2. **B** IP of endogenous BRD2 by transfected Fl-E was tested in the presence or the absence (vehicle) of the BET inhibitor JQ1. **C** A competition experiment similar to that described in (A) was performed, using the indicated constructions, in the presence (+) or the absence (–) of the HDAC inhibitor Trichostatin A (TSA). **D** The efficiency of TSA treatment was assessed by western blot (WB), revealing the levels of the acetylated form of histone H3 at K27 (H3K27Ac). A Ponceau S staining is shown as a loading control. 30 µg of total protein were loaded per lane. **E** The indicated HA-tagged constructs of BRD4 were transfected together with the Fl-E expression construct and cells were processed for IP with anti-Fl antibodies followed by WB with anti-HA antibodies. **A**–**C**, **E** 5% of each immunoprecipitation extract was loaded as input. **A**, **C**,** E** A schematic representation of the different constructions used is included in each corresponding panel. CTD, C terminal domain

As interaction with BET bromodomains was not only initially suggested [13], but also recently reported [17, 18], we further investigated BRD2-E protein interaction through complementary approaches. We first conducted a competition experiment on the capacity of Flag-E to precipitate endogenous BRD2. To this end, we overexpressed either the N-terminal half of BRD2 containing both bromodomains or the small C-terminal region containing the SEED, together with Flag-E. We observed that while overexpression of the SEED region completely abolished the precipitation of endogenous BRD2, overexpression of the bromodomains had no effect (Fig. 3A). Besides, the RFP-SEED fusion protein was also able to compete with the precipitation of endogenous BRD2 (Supplementary Fig. S2). We next reasoned that if bromodomains can recognize E, or acetylated E as it has been reported [17, 18], blocking acetyl groups recognition surface in bromodomains with anti-BET drugs should result in decreased interaction. The use of the JQ1 anti-BET drug demonstrated that BET inhibition did not impair interaction (Fig. 3B). Even more, it improves precipitation, probably due in part to enhanced solubilization of deeply attached BRD2 chromatin fraction. Finally, we wanted to analyze how important E acetylation was for the observed interaction. To this purpose, and given that E protein shares similarity with histones, we conducted immunoprecipitation experiments in the presence of Trichostatin A (TSA), an inhibitor of histone deacetylases (HDACs), which enhances global histone acetylation. The use of TSA did not alter the observed Flag-E-mediated precipitation of endogenous BRD2, nor the capacity of overexpressed BRD2 C-terminal half (containing the SEEDs) to compete for the interaction or the absence of effect of bromodomains in competition (Fig. 3C). In this experiment E was again able to precipitate the expressed C-terminal half of BRD2, but not the bromodomains. We checked the efficiency of TSA treatment by corroborating enhanced histone H3 acetylation at Lys 27 (H3K27ac) (Fig. 3D). Finally, since the works reporting the interaction of E with bromodomains have focused on BRD4, we wonder if there are peculiarities that differentiate the bromodomains of BRD2 from those of BRD4 that explain the different observations. Therefore, we tested the region spanning BRD4 bromodomains and the region from mB to SEED for interaction with E protein, and similar to BRD2, it was the region containing the SEED motif, and not the one containing the bromodomains, that strongly mediated the interaction of BRD4 with E (Fig. 3E).

### Overexpression of the E protein activates the interferon response

**Fig. 4:**
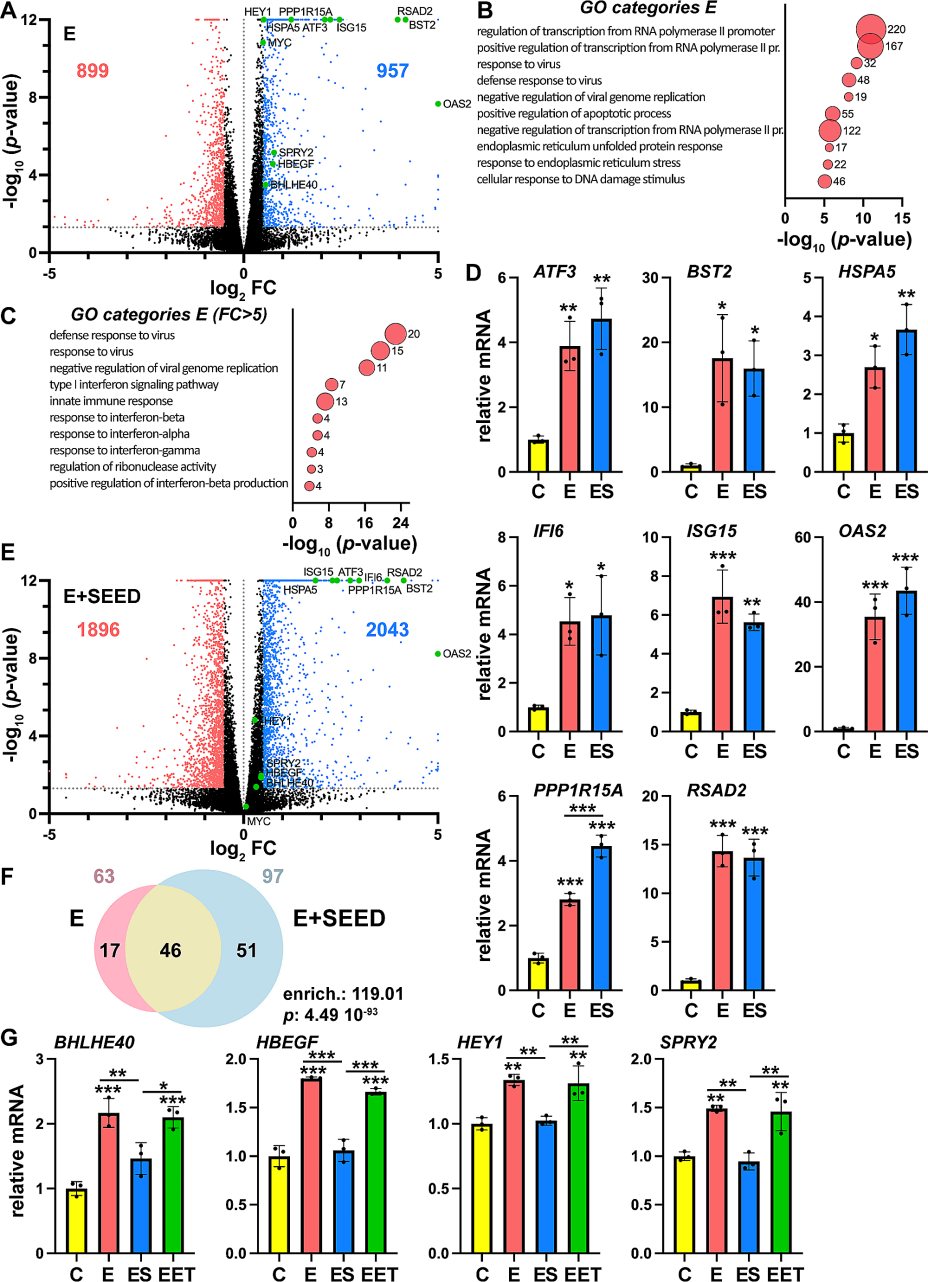
E overexpression induces the interferon response. **A** Volcano plot of genes misregulated by E overexpression upon RNA-seq analysis. Genes out of *p*-value and fold change (FC) cutoffs are in black. Downregulated genes are represented in red, while upregulated genes are represented in blue. Selected genes are highlighted in green. Numbers represent the number of misregulated genes in each category. **B**, **C** Gene ontology (GO) analysis of genes misregulated by E overexpression was represented by bubbles graphics, considering all misregulated genes (B) or only those with an FC > 5 (C). Bubble size represents the number of genes in each category, also indicated next to each bubble. *p*-value cutoffs of 10^− 5^ and 10^− 4^ were established for (B) and (C), respectively. **D** RNA-seq results were validated by quantitative PCR (qPCR) on a selection of genes. Relative mRNA levels of cells transfected with empty vector (control, C), with E expression construct (E), or with E and SEED expression constructs (ES), are represented. **E** Volcano plot of genes misregulated by combined expression of E and SEED upon RNA-seq analysis. See (A) for details. **F** Overlapping of genes misregulated by E alone or by combined E and SEED expression, with an FC > 5, is represented by a Venn diagram. Numbers on top of the diagram indicate the total number of misregulated genes in each condition. Enrichment (enrich.) of the overlapping and its associated *p*-value, determined by the hypergeometric test, are also indicated. **G** Expression of a selection of genes was analyzed by qPCR in the same conditions as in (D), and also after transfection of combined expression of E with ET (EET). **D**, **G** Bars indicate means ± s.d. of 3 independent experiments analyzed in triplicate. Statistical significance of changes in gene expression was analyzed by one-way ANOVA (*p* < 0.05) followed by Tukey’s post-test: * *p* < 0.05, ** *p* < 0.01, *** *p* < 0.001. Differences with control were indicated on top of each bar, other differences were indicated with a line

We next wondered about the effects on cells of overexpressing E. To this end, we decided to study the cell transcriptome by comparing cells transfected with an E expression construct with cells transfected with empty vector. Thus, samples for RNA-seq analysis were prepared in duplicate (see Supplementary Fig. S3A for checking of protein expression and Supplementary Fig. S3B for PCA). We initially considered changes with a *p*-value < 0.05 and a |log_2_ fold change (FC)| ≥ 0.5. We found 957 upregulated and 899 downregulated genes (Fig. 4A and Supplementary Table S1). Gene Ontology (GO) analysis of misregulated genes showed categories related to response to virus and to unfolded protein response/ER stress, besides categories related to transcription regulation (Fig. 4B). By looking at most upregulated genes (FC > 5) it came evident that top GO categories corresponded to response to virus, innate immune response and the interferon response (Fig. 4C). To confirm our RNA-seq results, we checked by reverse transcription and quantitative PCR (RT-qPCR) changes in expression of selected genes related to viral and unfolded protein responses (Fig. 4D). Consistent with RNA-seq, we observed upregulation of all the analyzed genes by E overexpression through RT-qPCR.

As we have determined that the SEED domain interacts with E protein, we also wondered whether SEED overexpression can annulate the effects of E overexpression. Then, we prepared duplicate samples for RNA-seq of cells transfected with expression vectors for E and SEED (Supplementary Fig. S3). Compared with the effect of E alone, a higher number of genes were misregulated with the addition of SEED (Fig. 4E and Supplementary Table S1), suggesting additional effects by SEED or combined expression of SEED with E. Interestingly, previously selected genes related to viral and unfolded protein responses showed similar transcription alterations as in the case of the overexpression of E alone (Fig. 4D). Indeed, a high overlap (85%) between genes misregulated by E alone and those misregulated in the combined presence of E and SEED was observed (Supplementary Fig. S4A), and much greater enrichment of overlapping was obtained when focusing on most upregulated genes (FC > 5) (Fig. 4F). For these genes, GO analysis showed again categories related to response to virus and the interferon response (Supplementary Fig. S4B), indicating that SEED does not block major effects of E overexpression. However, we observed a group of genes (284) only misregulated in the presence of E alone (Supplementary Fig. S4A), on which it is possible for SEED to counteract the effect caused by E. These genes are grouped into overlapping GO categories related to development and response to chemical or organic substances (Supplementary Fig. S4C). Data from RNA-seq showed that on these genes, SEED seems to annulate the effect of E on both upregulated and downregulated genes (Supplementary Fig. S4D). RT-qPCR analysis of selected genes confirmed this observation. By contrast, the expression of the ET domain, used as a control, was unable to annulate the effect of E (Fig. 4G).

On the other hand, we also analyzed GO categories associated with genes misregulated when overexpressing E together with SEED, but not misregulated by E alone. Most relevant categories were related to transcription regulation and intracellular transport of proteins (Supplementary Fig. S4E).

### BET inhibition does not mimic the effect of E overexpression

**Fig. 5:**
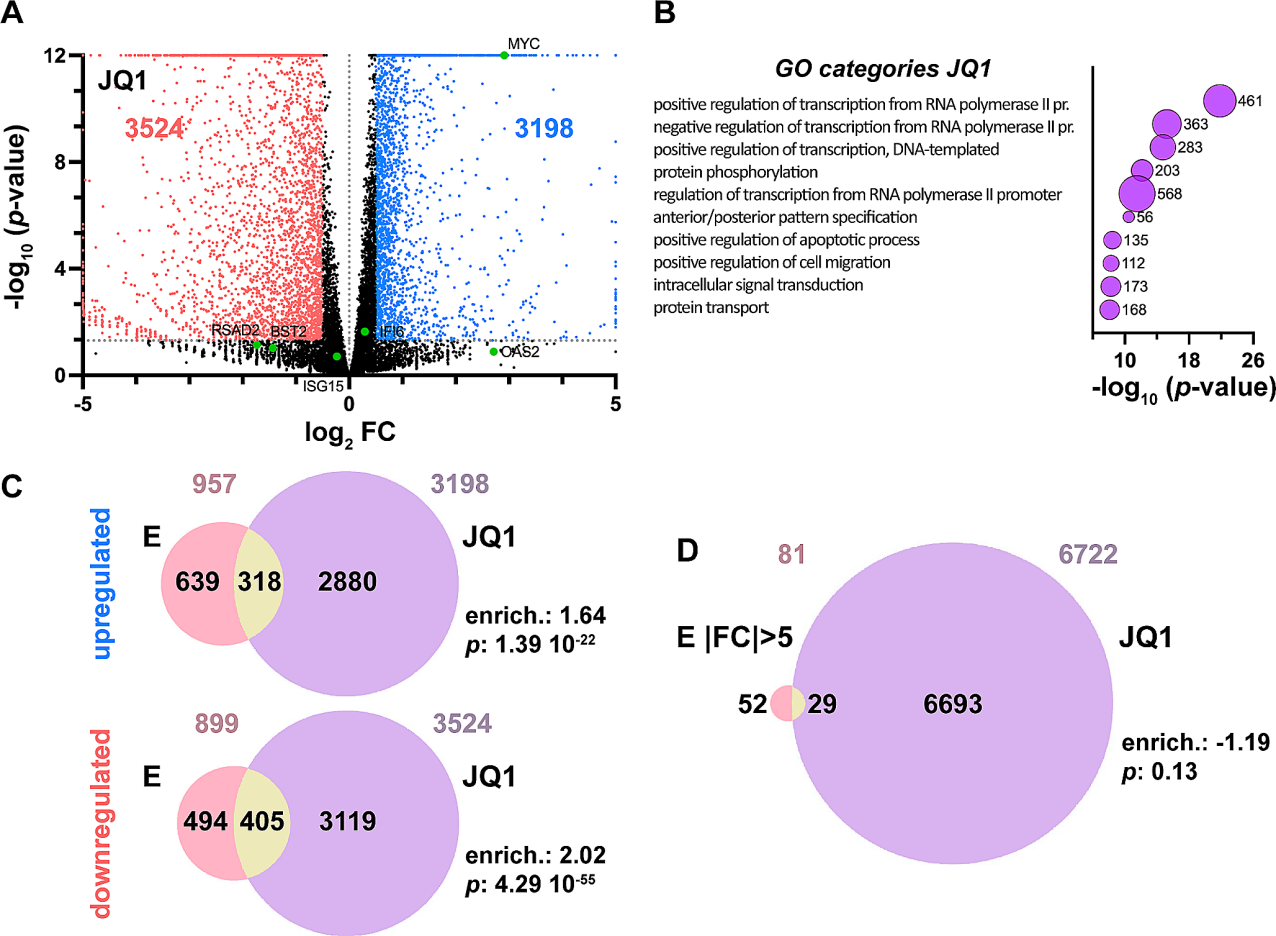
BET inhibition does not recapitulate E-mediated transcriptional effects. **A** Volcano plot of genes misregulated by BET inhibition with JQ1 drug upon RNA-seq analysis. See Fig. 4A legend for details. **B** Bubbles graphic of GO analysis of genes misregulated by JQ1 treatment. Bubble size represents the number of genes in each category, also indicated next to each bubble. A *p*-value cutoff of 10^− 8^ was established. **C** Venn diagrams representing the overlapping of genes upregulated and downregulated by E or BET inhibition (JQ1). **D** Venn diagram representing the overlapping of genes misregulated by E (only genes with a |FC| ≥ 5) or BET inhibition (JQ1). **C**, **D** Numbers on top of the diagrams indicate the total number of misregulated genes in each condition. Enrichment (enrich.) of the overlapping and its associated *p*-value, determined by the hypergeometric test, are also indicated

As the SARS-CoV-2 E protein interacts with BET members and it has been indicated that it may constitute a hijacking mechanism of the virus toward the host cell [13, 17, 18], we wondered whether the effect of E overexpression is comparable to BET inhibition. Therefore, we used the anti-BET drug JQ1 to treat cells for comparison of their transcriptome with that of cells overexpressing E (Supplementary Fig. S3B). We found that more than 6000 genes were affected by JQ1 treatment (Fig. 5A and Supplementary Table S1). As expected, among the most relevant GO categories of JQ1 misregulated genes, those related to transcriptional control stood out (Fig. 5B). When comparing genes misregulated by JQ1 with those misregulated by E overexpression we observed some overlapping (33% and 45% of genes upregulated and downregulated by E, respectively), associated with a modest enrichment (Fig. 5C). However, when comparing the most misregulated genes by E overexpression (|FC| > 5), among which are most of the genes related to response to virus, the innate immune response and the response to interferon, with the genes misregulated by JQ1, no enrichment was observed at all (Fig. 5D). This indicates that JQ1 and E overexpression lead to different transcriptional outcomes.

**Fig. 6:**
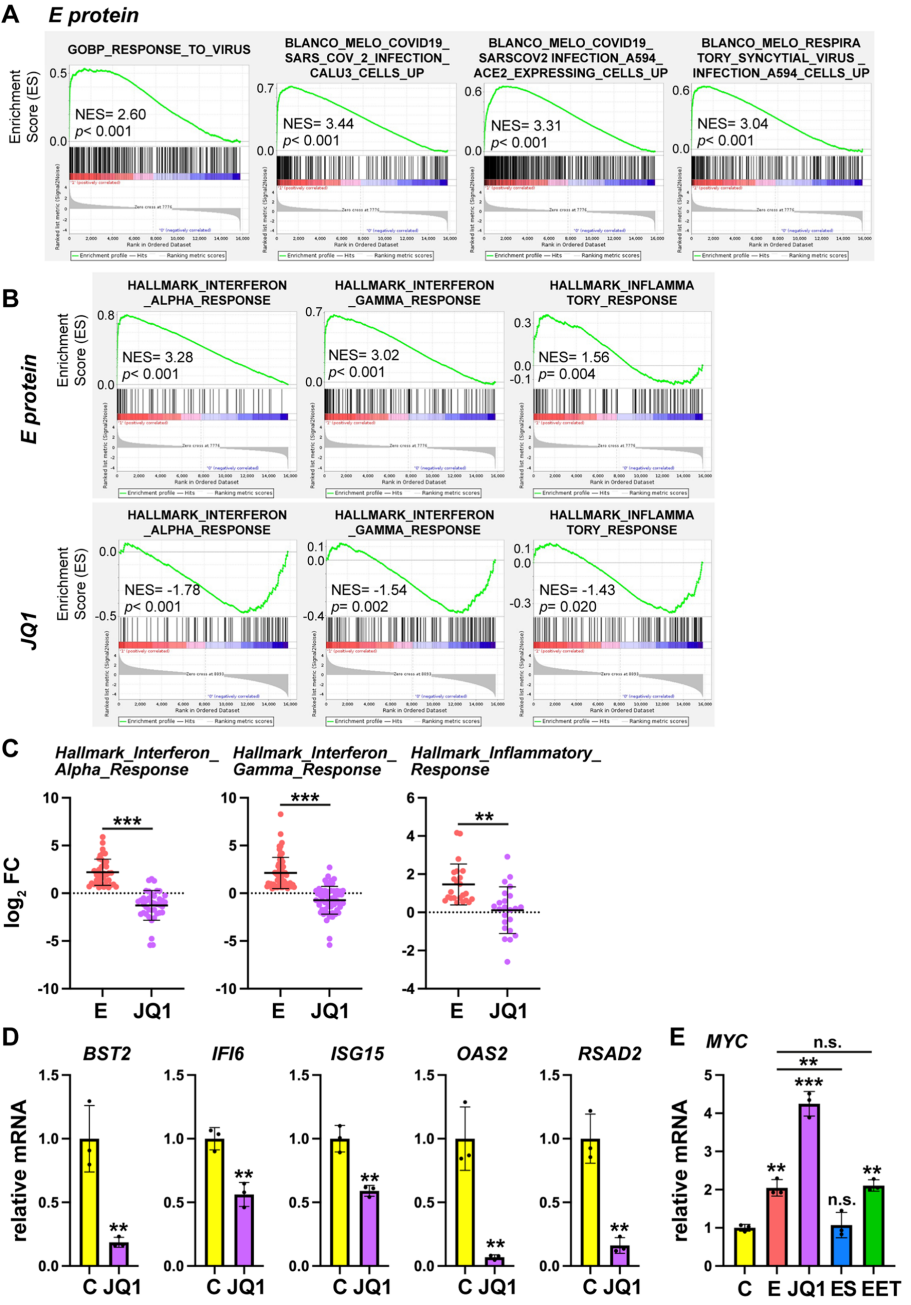
Opposite effects of BET inhibition and E overexpression on the immune response. **A** Gene Set Enrichment Analysis (GSEA) plots from the analysis of RNA-seq data of misregulated genes after overexpression of E protein. Some significative categories are shown. Normalized enrichment score (NES) related to aleatory samples of the same size is shown for each plot, as well as the statistical significance for the enrichment through the nominal *p*-value. **B** Comparison of GSEA plots from the same selected categories derived from RNA-seq data of cells overexpressing E protein and cells treated with the BET inhibitor JQ1. See (A) for details. **C** Representation of log_2_ FC values of misregulated genes in the different categories shown in (B) for comparison of E and JQ1 effects. A mean value ± s.d. for each set of genes under the different conditions is shown. **D** Effect of JQ1 drug in comparison with vehicle (control, C) on mRNA levels of immunity-related genes analyzed in Fig. 4D was assessed by qPCR. **E** Changes in mRNA levels of *MYC*, determined by qPCR, in comparison with control conditions (C, transfection of empty vector or vehicle), by overexpression of E alone (E), E together with SEED (ES) or E together with ET (EET), or after treatment with JQ1. **D**, **E** Bars indicate means ± s.d. of 3 independent experiments analyzed in triplicate. **C**-**E** Statistical significance was determined by paired (C) or unpaired (D) Student’s *t*-test, or by one-way ANOVA (*p* < 0.05) followed by the Tukey’s post-test (E): n.s. not significative, ** *p* < 0.01, *** *p* < 0.001. Differences with control were indicated on top of each bar, other differences were indicated with a line

For a more detailed comparison, we performed a Gene Set Enrichment Analysis (GSEA) of JQ1 and E misregulated genes. Remarkably, genes upregulated by E overexpression were strongly enriched in gene sets related to viral infection. It especially caught our attention categories related to the response of different cell types to infection by SARS-CoV-2 or other viruses (Fig. 6A). A thorough comparison of our results with previously published data [24] indicated that the overexpression of the E protein significantly recapitulates the transcriptome changes caused by SARS-CoV-2 virus infection in Calu-3 cells (Supplementary Fig. S5A). Thus, 30% (*p*-value of the hypergeometric test: 3.38 10^− 45^) of the genes upregulated by SARS-CoV-2 infection in Calu-3 cells were among the genes upregulated by E in HEK293T cells. Considering the most upregulated genes in HEK293T cells (FC > 5), 51% (*p*-value of the hypergeometric test: 4.61 10^− 37^) of them were also upregulated by SARS-CoV-2 infection in Calu-3 cells (Supplementary Fig. S5A). Consistently, gene sets related to the inflammatory response, interferon (IF) α and γ responses, and tumor necrosis factor A (TNFA) signaling, were also very significantly enriched among genes upregulated by E (Fig. 6B and Supplementary Fig. S5B). In sharp contrast, JQ1 caused a general downregulation of inflammatory response and IFα and IFγ responses gene sets (Fig. 6B). Indeed, the representation of RNA-seq data corresponding to genes grouped in these categories confirmed this observation (Fig. 6C). As observed, most genes upregulated by E inside these categories, were downregulated or not regulated by JQ1. JQ1-mediated downregulation of infection-related genes analyzed in Fig. 4D was corroborated by RT-qPCR (Fig. 6D). GSEA also showed that genes misregulated by E overexpression were enriched in the unfolded protein response gene set (Supplementary Fig. S5B), while exclusive gene sets related to BET functions [25] were associated with JQ1 treatment and not with E overexpression (Supplementary Fig. S5B).

Interestingly, we also observed a GSEA category of misregulated genes with similar plots for both E overexpression and JQ1 treatment: MYC targets (Supplementary Fig. S5C). This caught our attention because *MYC* gene is a classical target of BETs [26]. We realized that indeed it is *MYC* gene that was misregulated, so its targets can be affected. Both E and JQ1 upregulated *MYC* expression and interestingly SEED, but not ET, was able to antagonize the E effect (Fig. 6E). In parallel, *MYC* targets were also upregulated on average by JQ1 and to a lesser extent by E (Supplementary Fig. S5D). However, they were upregulated in a more modest manner than the *MYC* gene itself (Fig. 6E).

### SEED-specific interaction of E can be used for its targeted degradation

**Fig. 7:**
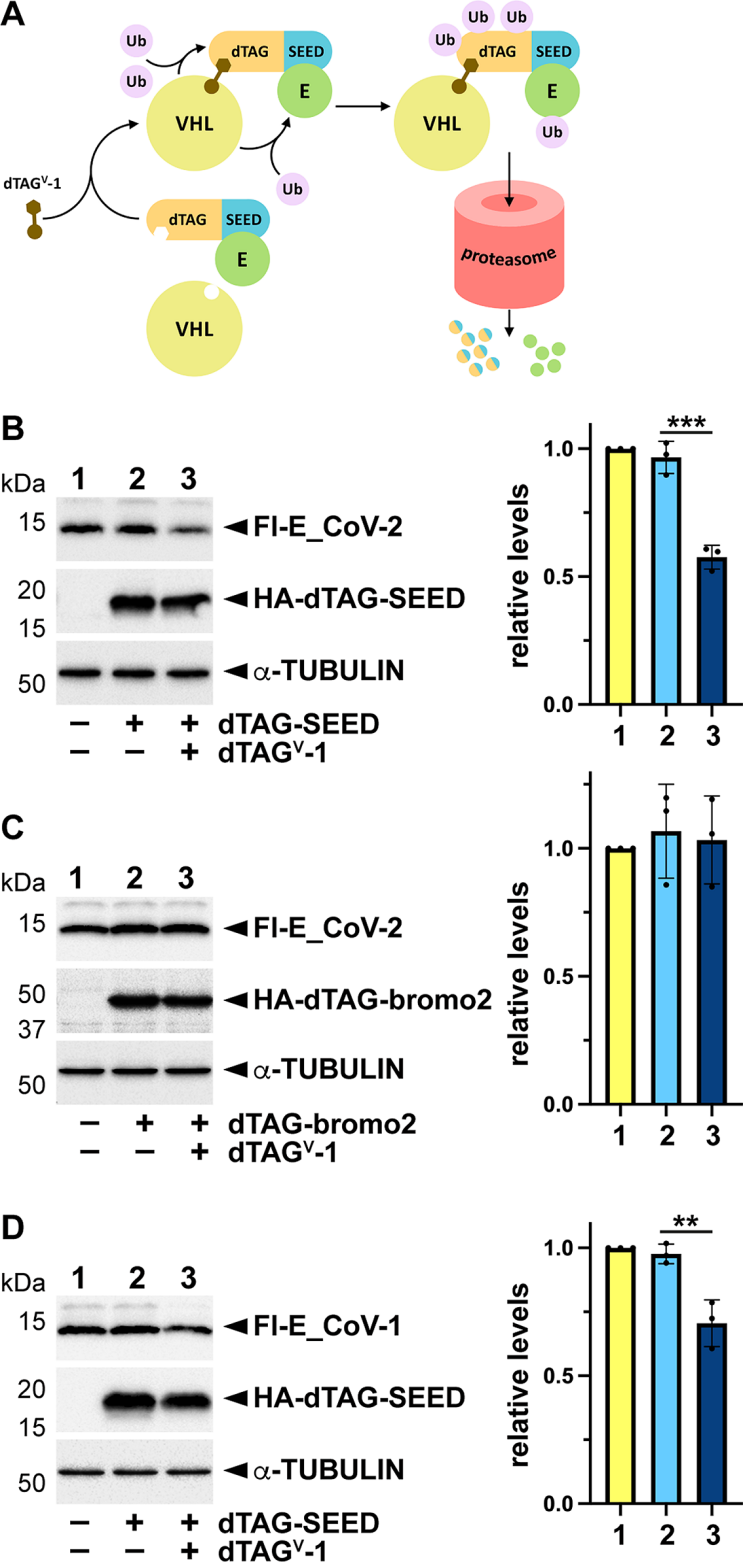
A dTAG-SEED fusion protein can mediate E protein degradation. **A** Schematic representation of the working hypothesis. The dTAG-SEED fusion protein, through the PROTAC dTAG^V^-1, recruits the Von Hippel-Lindau (VHL)-associated Ubiquitin (Ub) E3 ligase complex to ubiquitylate the fusion protein, but side ubiquitylation of the interacting E protein can also occur, which promotes proteasome-mediated degradation of both proteins. **B** HEK293T cells were transfected with the SARS-CoV-2 Flag (Fl)-E expression construct alone or in combination with the HA-dTAG-SEED (BRD2 amino acids 771–798) expression construct as indicated. **C** HEK293T cells were transfected with the SARS-CoV-2 Fl-E expression construct alone or in combination with the HA-dTAG-bromo2 (BRD2 amino acids 171–472) expression construct as indicated. **D** HEK293T cells were transfected with a Fl-tagged expression construct involving the SARS-CoV-1 E protein alone or in combination with the HA-dTAG-SEED expression construct as indicated. **B**-**D** Cells expressing both proteins were treated (+) or not (–, vehicle: DMSO) with the PROTAC dTAG^V^-1 for 24 h and subjected to western blot (left) to estimate the relative levels of Fl-E protein in each condition (right). Relative levels, normalized to lane 1, are represented. Values are means ± s.d. of 3 independent experiments. Statistical significance of differences between conditions were analyzed by one-way ANOVA (*p* < 0.05) followed by Tukey’s post-test: ** *p* < 0.01, *** *p* < 0.001

Since E interaction with BETs seems to be mediated by the SEED domain, a small region of a few amino acids, we wondered about the possibility of exploiting this feature to direct targeted degradation of E protein. To this purpose, we took advantage of the dTAG PROTAC-based degron system, which uses mutant FKBP12 (F36V) protein as a degron tag [27]. This tag, fused to a protein of interest, in the presence of specific PROTAC chemicals mediates the recruitment of particular ubiquitin E3 ligase complexes, which leads to the ubiquitylation of the fusion protein and degradation by the proteasome [28]. However, when the target protein forms a complex with others, side ubiquitylation in the interactors can also occur (Fig. 7A). Thus, we built a dTAG-SEED fusion construct and we transfected HEK293T cells with Flag-E expression construct only, or together with the dTAG-SEED construct. Then, cells expressing both constructs were treated with the PROTAC dTAG^V^-1 and interestingly, after 24 h, we observed a significant reduction of E protein levels in comparison with non-treated cells (vehicle) (Fig. 7B). As a control we used a dTAG-bromo2 construct, including the second bromodomain of BRD2, expected not to interact with E, which resulted in unaltered levels of E after treatment with the PROTAC (Fig. 7C). We also wondered if the SARS-CoV-1 E protein, interacting with BRD2 as well, was affected by the PROTAC. As shown in Fig. 7D, and similarly to SARS-CoV-2 E, SARS-CoV-1 E levels were reduced in the assay.

## Discussion

Because the SARS-CoV-2 E protein has been reported to interact with BET members, we aimed to study the consequences of this interaction to evaluate whether it constitutes a virus-hijacking mechanism as previously proposed. To get insight into the molecular mechanism underlying this phenomenon, we first decided to precisely map the interaction surfaces of the proteins, and then to compare the gene expression pattern displayed by cells overexpressing E protein with that of cells treated with an inhibitor of BET proteins.

Surprisingly, and differing from previous reports pointing to the BET bromodomains as responsible for E interaction [17, 18], we have found that it is a SEED-like region in BRD2 and the SEED domains of BRD2 and BRD4 that mediate interaction. Several approaches support our conclusion: (i) immunoprecipitation assays with deletion constructs indicate this. E protein is unable to precipitate the N-terminal half of BRD2 encompassing both intact bromodomains, but the addition of a small fragment corresponding to a SEED-like region (Ac domain) leads to immunoprecipitation. Similarly, a fragment containing the ET domain is not precipitated by E, but adding just the SEED enables precipitation. Moreover, a deletion construct lacking just the SEEDs together with the mB does not interact, but just adding the C-terminal SEED (28 amino acids) enables interaction; (ii) the SEED domain, in a small C-terminal fragment, and just the SEED fused to RFP are efficiently precipitated by E; (iii) most of the interactors of E protein reported by Gordon and co-workers [13] (5 out of 6, including BRD2 and BRD4) contain SEED-like domains. Indeed, those of BRD4 and AP3B1, fused to RFP are also efficiently precipitated by E; (iv) competition experiments evaluating precipitation of endogenous BRD2 by E, also support our findings. Thus, the SEED domain fused to RFP and different C-terminal fragments containing the SEED, strongly impair precipitation of endogenous BRD2, while a construction encompassing both bromodomains has no effect, even under high acetylation conditions. Of note, the SEED-like domain (Ac region) has been previously demonstrated to constitute a region for protein interaction [5].

In those reports describing E interaction with the BET bromodomains, experiments are performed with acetylated E protein [17, 18]. However, we have also conducted immunoprecipitation assays under high acetylation conditions and observed once again that bromodomains are not precipitated by E and that they are not competing for the precipitation of endogenous BRD2. JQ1 drug mimics acetyl groups and thereby blocks the ability of BETs to recognize acetylated proteins. Thus, the absence of JQ1-mediated effect on E-driven precipitation of endogenous BRD2 also supports the notion that bromodomains are expendable for interaction. As our interaction analysis mostly relied on BRD2, while previous work on E-BET interaction has been essentially conducted on BRD4 [17, 18], we also tested the interaction of E with BRD4 bromodomains and with the region containing the SEED, corroborating the same observations realized on BRD2. Moreover, we have demonstrated E interaction with isolated BRD4 SEED domain. On the other hand, it has also been reported that another interaction region of BETs with E is the ET domain [18], a region usually targeted by different viruses on BET members [11, 12]. However, in our immunoprecipitation experiments, we have not detected such interaction. A possible issue concerns the exact definition of the ET domain, located close to the SEED, so in some studies, it partially or totally includes the SEED [29, 30]. In any case, we cannot exclude that divergent observations are explained by differences in procedures or cells used.

Regarding E protein, we have mapped the interaction surface with SEED to the intra-virion region. However, the previously described protein-protein interaction domain at the C-terminus of E, the PBM, was dispensable for the interaction. This motif has been demonstrated to interact with PDZ domains of PALS1 and ZO1 proteins, involved both in tight junctions formation and integrity in epithelial cells [22, 23]. Instead, a preceding 15-amino acids stretch was necessary for E interaction. Interestingly, this stretch contains the residues that were initially observed to differentiate E proteins of SAR-CoV-1 and SARS-CoV-2. However, differences do not result in altered interaction, as we have also shown the interaction of BRD2 with SARS-CoV-1 E.

We have observed a marked effect of E overexpression on HEK293T transcriptome. Previously, E overexpression was indicated not to lead to major changes in cell transcriptome [31], which might be explained by low overexpression levels and/or cell lines used in previous studies. In agreement with protein overexpression, we have observed activation of genes related to the unfolded protein response and ER stress. Indeed, ER/Golgi has been proposed as a major site of E protein localization [15, 16]. Nevertheless, the most outstanding observation was the activation of the interferon response. Indeed, the majority of most upregulated genes (FC ≥ 5) were related to this. This is in agreement with a previous report demonstrating the induction of reactive oxygen species (ROS) generation and inflammatory signaling by purified SARS-CoV-2 surface proteins E and S (Spike), directly and independently of virus infection [32]. Moreover, the E protein has been demonstrated to be recognized by the TLR2 receptor, which leads to the production of inflammatory cytokines [33, 34]. Surprisingly, overexpression of a BRD2 fragment containing the E interacting motif (SEED), was not able to antagonize activation of the interferon response. Since it has been described that capacity of E protein to induce the generation of ROS and inflammation resides in its 10 N-terminal amino acids [32], and since we have mapped interaction surface with SEED to the intra-virion region located at the C-terminal half of E, it is plausible that SEED interaction is not blocking N-terminal activity of E. Alternatively, induction of the interferon response might also rely, at least in part, on E mRNA derived from our expression construct, as SARS-CoV-2-associated single strand RNAs have been also implicated in activation of inflammation and immunity through TLR7/8 [35, 36].

On the other hand, we wondered about the interference of E with BET function. To this purpose, we compared the effect of E overexpression with that of BET inhibition. Our results demonstrated opposite effects on the natural immune response. Many of the genes upregulated by E, associated with the interferon response, were downregulated by BET inhibition. This agrees with previous works highlighting the requirement of BET function (especially of BRD4) for the immune response (revised in [10]), and it also agrees with the previously reported effect of purified E in inducing interferon-mediated inflammation [32]. It was initially proposed to use BET inhibition to treat COVID-19, based on the requirement of BETs for the expression of the host ACE2 receptor, which mediates viral entry [31, 37, 38]. BET inhibition has also been indicated to be useful in hampering the exacerbated immune response associated with SARS-CoV-2 infection and linked to severe tissue damage [39, 40]. However, our results and those from Anand and co-workers [32] differ from other results indicating that E phenocopies the suppression of interferon production driven by BET inactivation [17, 18]. We observe that E overexpression is not significantly affecting BET function, or at least not to the same extent as anti-BET drugs do, which does not support E interaction with BETs as a virus-hijacking mechanism. We have indeed observed some overlapping between E- and JQ1-misregulated genes. Given the great number of genes affected by JQ1, such an overlapping is predictable. However, we have found in particular some genes, related to development and response to chemical or organic substances, modestly altered by E, whose effect can be antagonized by SEED, suggesting that for this set of genes, E may display some BET-associated effect titrated and released by SEED overexpression. This set of genes might depend to some extent on SEED for regulation, but not notably, which might explain the milder transcriptional effects observed. This is consistent with the fact that SEED has not been involved to date in transcription as, for instance, the bromodomains. Another gene behaving similarly was *MYC*, a classical BET target described to be upregulated by BET inhibition in several cell lines [41, 42]. Indeed, as for BET inhibition, but to a lesser extent, we have observed E-mediated upregulation of *MYC*. Once again full and accurate *MYC* regulation might also depend, at least in part, on the SEED domain.

Finally, we moved on to exploit the specific interaction of the E protein with SEED for targeted degradation of E. Our results indicate that the use of a PROTAC-based degron system may lead to the degradation of E protein from both SARS-CoV-2 and SARS-CoV-1, which opens new and promising perspectives in fighting COVID-19 and possibly other infectious diseases. To our knowledge, no other interactors of the SEED domain have been described to date. We have previously reported the interaction of the secreted growth factor pleiotrophin (PTN) with the SEED-like motif (Ac region) of BRD2 but not with the SEED domain [5]. In addition, the roles of PTN are mainly linked to the development and safeguarding of the nervous system [43, 44], so its expression is normally associated with particular processes and probably occurs in a cell type-restricted manner. Thus, the interaction of E protein with SEED appears highly specific, which constitutes an important advantage for its therapeutic exploitation as a system targeting E for selective degradation through SEED fusions with appropriate degradation TAGs.

## Materials and methods

### Plasmid constructs

RFP expression constructs were derived from plasmid pDsRed-Monomer-Hyg-C1 (Clontech-TAKARA, San Jose, CA, USA), introducing an HA tag at the N-terminus of the encoded proteins. The rest of expression constructs were based on the pAdRSV-Sp vector [2], with N-terminal HA or Flag tags. HA-BRD2 and HA-BRD4 expression constructs have been previously described [2]. Deletion and domain constructs derived from these proteins have been partly described in [2, 5], or obtained by standard PCR techniques, amplifying a DNA fragment corresponding to the indicated amino acids and cloned between *Nhe*I and *Nsi*I sites of pAdRSV-Sp. The cDNAs for E protein and dTAG were obtained from plasmids pCAGGS-E [16] and pLEX_305-C-dTAG (a gift from James Bradner and Behnam Nabet: http://n2t.net/addgene:91798, Addgene plasmid # 91798), respectively. The cDNA for SARS-CoV-1 E was obtained from SARS-CoV-2 E cDNA by PCR-mediated mutagenesis. The cDNA for the AP3B1 SEED domain was obtained by retrotranscription of total RNA from HEK293T cells and PCR amplification with primers: forward 5’-AGAAGCTAGCTTCTGAATCTGAGGAAGAGGAGG-3’ and reverse 5’-GGCCATGCATCTATCCACTCTCACTGTCCTGCTCAC-3’.

### Cell culture and transfection

Human HEK293T cells were cultured in Dulbecco’s Modified Eagle’s Medium (DMEM) (Sigma-Aldrich, St. Louis, MO, USA) supplemented with 10% fetal bovine serum (Sigma-Aldrich) and with 10 ml/l of an antibiotic solution with Penicillin (100 U/ml) and Streptomycin (10 mg/ml) (Sigma-Aldrich). Transfections were performed with Lipofectamine 2000 (Invitrogen, Life Technologies, Paisley, UK) for 24 h, following the manufacturer’s recommendations.

### BET inhibition, deacetylase inhibition, histone extraction, and dTAG procedures

For BET inhibition, JQ1 drug (Sigma-Aldrich) was used at 0.5 µM for 16 h, both in competition experiments and for transcriptomic analysis. To procure increased protein acetylation, trichostatin A (TSA) (Sigma-Aldrich), was used at 1.65 µM for 4 h before harvesting the cells. For histone extraction, buffer [5 mM butyric acid, 0.5% Triton X-100, in phosphate-buffered saline (PBS)] was used at 4 °C under rotation for 10 min, then, the extract was centrifuged, and the resulting pellet resuspended in 0.2 N HCl for overnight incubation at 4 °C under rotation. After a new centrifugation, the supernatant was conserved and neutralized with NaOH for western blot analysis. For PROTAC treatment, cells were normally transfected, and 6 h after transfection, the PROTAC dTAG^V^-1 (TOCRIS Bioscience, Bristol, UK) from a 10 mM stock in DMSO was added at a final concentration of 2 µM and maintained for 24 h before cell harvesting.

### Immunoprecipitation and western blot

For immunoprecipitation experiments, cells were extracted with buffer [50 mM Tris-HCl pH 7.5, 1% Triton X-100, complete protease inhibitor cocktail with EDTA (Roche, Mannheim, Germany)], supplemented with 540 mM NaCl, and diluted to 150 mM before the addition of antibodies. Protein concentration was determined by the Bradford reactive assay (Bio-Rad, Hercules, CA, USA) and 0.5 mg of total protein was incubated overnight at 4 °C in rotation with 3 µg of the corresponding antibody or normal mouse or rabbit IgG (Sigma-Aldrich) as control. Antibodies were precipitated by incubation under rotation with protein A (rabbit-raised antibodies) or G (mouse/rat-raised antibodies) Dynabeads (ThermoFisher Scientific, Waltham, MA, USA) at 4 °C for 2 h. After washing, proteins were eluted from beads with 20 µL of Laemmli buffer and 3 min of boiling to be analyzed by immunoblotting. For this, eluted proteins or whole extracts (25 µg of total protein) were separated in SDS gels and transferred to PVDF membranes (GE Healthcare) for blotting with antibodies. The membrane was processed with a chemiluminescence ECL system (Bio-Rad) and analyzed in a ChemiDoc XRS apparatus (BioRad). Antibodies used in western blots were: mouse anti-FLAG M2 (1:1000, #F1804, Sigma-Aldrich), rabbit anti-HA (1:1000, #H6908, Sigma-Aldrich), mouse anti-α-TUBULIN (1:2000, # T9026, Sigma-Aldrich), rabbit anti-BRD4 (1:1000, #A301-985A100, Bethyl Laboratories, Inc., Montgomery, TX, USA), rabbit anti-BRD2 serum (1:500, [2]), rabbit anti-H3K27Ac (1:1000, #ab4729, Abcam, Cambridge, UK) and horseradish peroxidase (HRP)-conjugated goat anti-mouse and goat anti-rabbit IgG (1:10000, Sigma-Aldrich).

### Proximity ligation assay (PLA)

For PLA, we used the Duolink PLA in situ detection reagents red kit (Merck, Darmstadt, Germany), following the manufacturer’s recommendations. Cells were fixed in 4% paraformaldehyde for 10 min and then permeabilized with 0.5% Triton X-100 in PBS buffer for 5 min. Rabbit anti-BRD2 serum (1:100, [2]) and mouse anti-FLAG M2 (1:100, #F1804, Sigma-Aldrich) were used as primary antibodies, coupled to anti-rabbit (plus) and anti-mouse (minus) Duolink in situ PLA probes (Merck). Negative controls were performed by omitting one or the other primary antibody from the assay.

### RNA extraction, retrotranscription, and quantitative PCR (RT-qPCR)

Total RNA was extracted from HEK293T cells using the NZY Total RNA isolation kit (NZYTech, Lisbon, Portugal) and retrotranscription was performed with the High Capacity cDNA Reverse Transcription Kit (Applied Biosystems, Carlsbad, CA, USA). qPCR was performed on total RNA with Power SYBR Green (Applied Biosystems) using a ViiA7 Real-Time PCR System (Applied Biosystems). The *RPLP0* gene was used as a reference gene to analyze relative expression. Normalization was done according to [45]. Data were derived from three independent experiments analyzed in triplicate. Used primers are described in supplementary Table S2.

### RNA-seq

Total RNA was extracted using the RNeasy kit (QIAGEN, Austin, TX, USA) and processed in CABIMER Genomics facility. Duplicate samples were prepared for each condition. The libraries were prepared using the TruSeq Stranded TOTAL RNA kit (Illumina, San Diego, CA, USA) and sequencing was accomplished with the NextSeq500 HIGH-Output and 1 × 75 bp length parameters. Obtained data were primarily filtered using the FASTQ Toolkit v1.0.0 program. Then, data were aligned using Subjunc function from Rsubread [46] v.1.28.1 bioconductor package, to map reads to the hg19 human reference genome, using TH1 = 2 and unique = TRUE parameters. The downstream analysis was performed on bam files. Duplicates were removed using the samtools v.0.1.19 rmdup command [47]. FeatureCounts() function from Rsubread v.1.28.1 bioconductor package was utilized to assign reads to UCSC hg19 KnownGenes using GTF.featureType="exon” and GTF.attrType="gene_id” parameters. Then, differential gene expression analysis was performed using the DESeq2 [48] from Bioconductor packages. Genes that were expressed at > 0.5 counts per million mapped reads in ≥ 2 replicates were analyzed. Principal component analysis (PCA) was performed using plotMDS from Limma-voom (v.3.34.9) [49] using CPM normalized reads. For each comparison (control vs. E overexpression, control vs. E and SEED overexpression, and control vs. JQ1 treatment) we selected those genes that were upregulated or downregulated with a *p*-value < 0.05 and |log_2_(FC)| ≥ 0.5.

### Statistical analysis and additional in silico tools

Statistical analyses were performed with the Prism 9.5.1 software (GraphPad). Mean values ± s.d. were represented in the different graphs. Two-tailed Student *t*-test or one-way ANOVA (*p* < 0.05) followed by the Tukey post-test for multiple comparisons were applied for statistical analysis of two groups (comparison with control) or more than two groups, respectively (*p* < 0.05*, *p* < 0.01**, *p* < 0.001***). Normal distribution and similar variances were assumed. To test the significance of overlapping in Venn diagrams, hypergeometric tests were performed at https://systems.crump.ucla.edu/hypergeometric/index.php. Venn diagrams were performed in Venny 2.1 (http://bioinfogp.cnb.csic.es/tools/venny/index.html) and drawn to scale with https://www.meta-chart.com/venn#/display. Gene ontology (GO) functional categories were analyzed using DAVID [50]. GSEA was performed using the GSEA v2.0.14 software (GSEA, Broad Institute, Cambridge, MA, USA) with 1,000 phenotype permutations [51].

### Electronic supplementary material

Below is the link to the electronic supplementary material.

Supplementary Material 1

Supplementary Material 2

## Data Availability

The datasets generated during the current study are available in the NCBI GEO repository and are accessible with the accession number GSE245466.
