# Supplementary material for: SARS-CoV-2 E protein interacts with BRD2 and BRD4 SEED domains and alters transcription in a different way than BET inhibition
Source: Cell Mol Life Sci. 2024 Jul 27;81(1):313. doi: 10.1007/s00018-024-05343-8 (PMC11335234; doi:10.1007/s00018-024-05343-8)
Supplement: Supplementary file 2 — Supplementary Material 2 [file 18_2024_5343_MOESM2_ESM.pdf]

Supplementary Material

SARS-CoV-2 E protein interacts with BRD2 and BRD4 SEED domains and alters transcription in a different way than BET inhibition

Nieves Lara-Ureña, Elena Gómez-Marín, Isabel Pozuelo-Sánchez, Jose C. Reyes, Mario García-Domínguez

Supplementary Figures and Supplementary Table S2

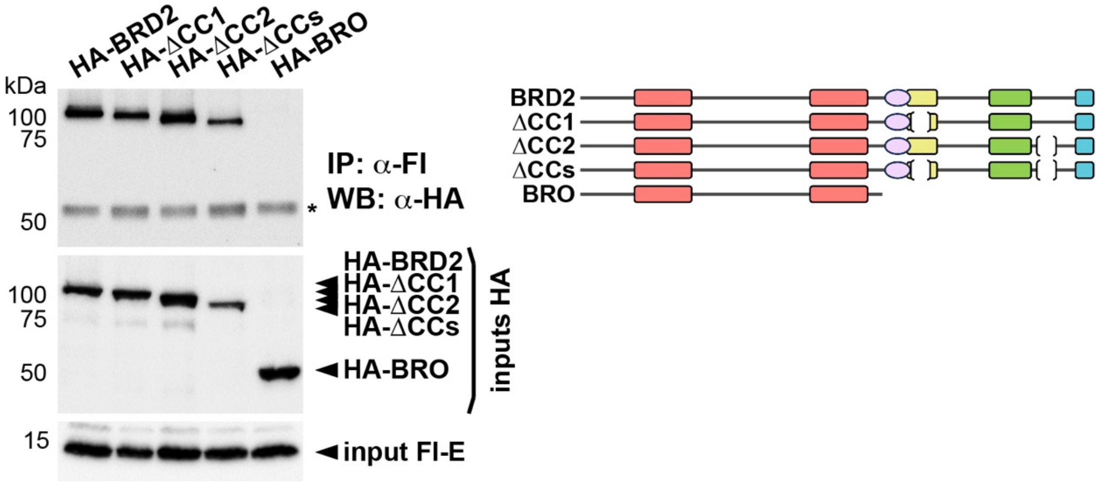

Supplementary Fig. S1 *BET coiled coils are not involved in the interaction with E.*

Different HA-tagged constructs were transfected with the Flag (Fl)-E construct in HEK293T cells for immunoprecipitation (IP) with anti-Fl antibodies followed by anti-HA western blot (WB). A schematic representation of the different constructions used is included in the panel. CC, coiled coil. 5% of each immunoprecipitated extract was loaded as input. * IgG bands.

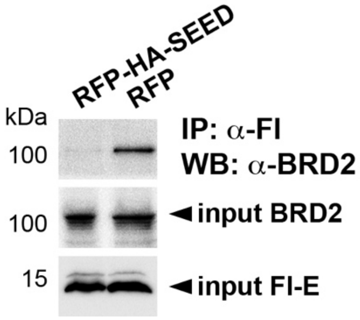

Supplementary Fig. S2 *SEED domain competes E interaction with endogenous BRD2.*

Flag (Fl)-tagged E was expressed in HEK293T cells either with the Red Fluorescent Protein (RFP) alone as with HA-tagged SEED fused to RFP, to test by western blot (WB) the capacity of these proteins to interfere with Fl-E-mediated immunoprecipitation (IP) of endogenous BRD2. 5% of each immunoprecipitated extract was loaded as input.

A

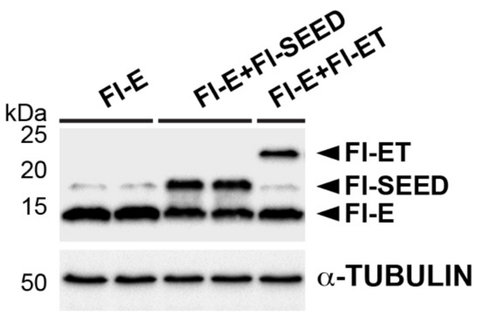

B

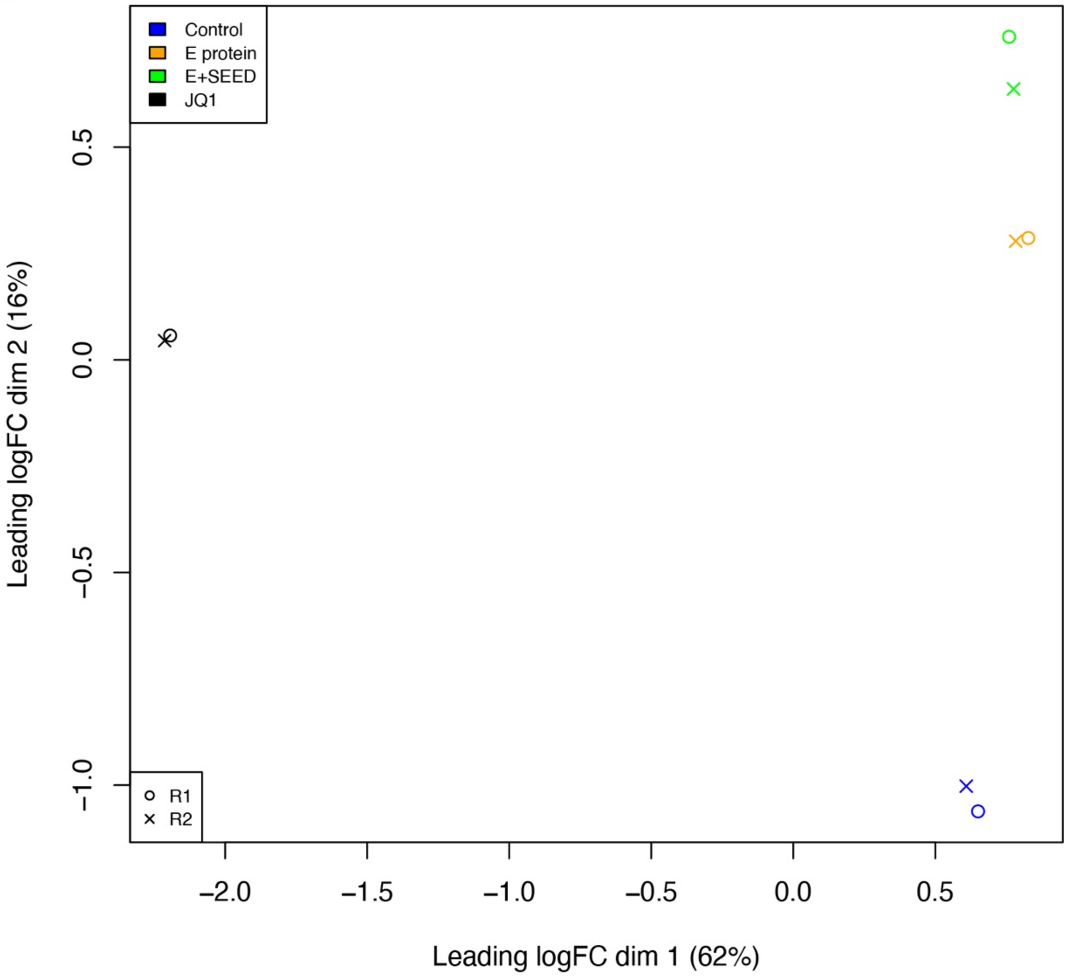

Supplementary Fig. S3 *Expression constructs and principal component analysis (PCA) representation related to transcriptomic analysis.*

A The different combinations of Flag (Fl)-tagged proteins expressed for RNA-seq analysis (in duplicate, lanes 1-4) or RT-qPCR experiments (lane 5) were revealed by western blot with anti-Fl antibodies. α-TUBULIN was also revealed as a loading marker. **B** PCA representation of the RNA-seq analysis. Sample replicates (R1 and R2) are indicated in the same color, with different symbols.

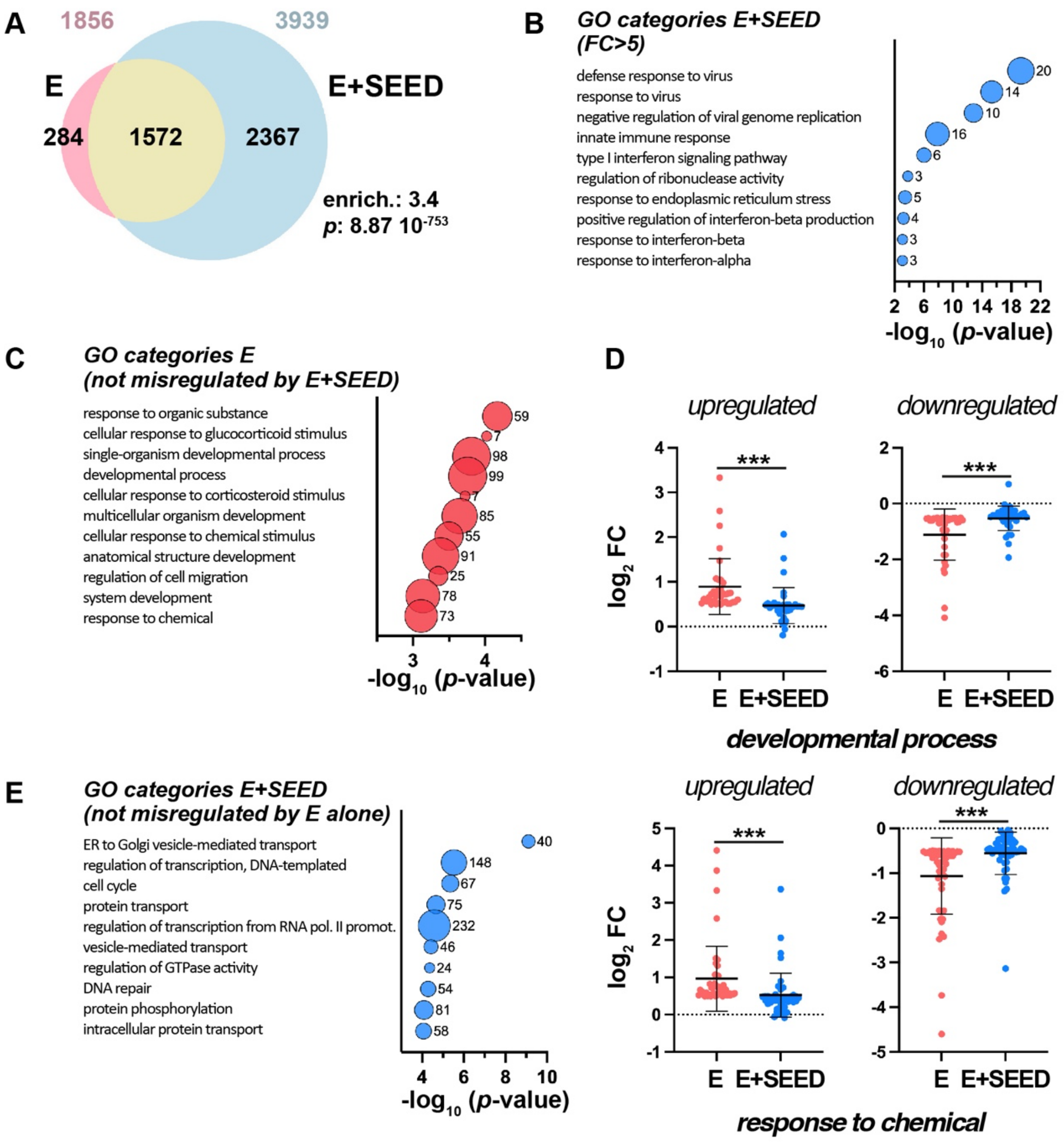

Supplementary Fig. S4 *Transcriptional effects mediated by E and SEED expression.*

A Overlapping of genes misregulated by E alone and by combined expression of E and SEED, represented by a Venn diagram. Numbers on top of the diagram indicate the total number of misregulated genes in each condition. Enrichment (enrich.) of the overlapping and its associated *p*-value, as determined with the hypergeometric test, are also indicated. **B** Bubbles graphic representation of gene ontology (GO) analysis of genes misregulated by combined expression of E and SEED with a fold change (FC) > 5 (*p*-value cutoff: $9{\cdot}10^{-4}$). **C** Bubbles graphic representation of GO analysis of genes misregulated by E that are not misregulated by combined expression of E and SEED (*p*-value cutoff: $8{\cdot}10^{-4}$). **D** Representation of $\log_2$ FC values of upregulated and downregulated genes in the GO categories indicated by E overexpression or combined expression of E and SEED. A mean value ± s.d. for each set of genes under the different conditions is shown. Statistical significance was determined by paired Student's *t*-test: *** $p < 0.001$. **E** Bubbles graphic representation of GO analysis of genes misregulated by combined expression of E and SEED that are not misregulated by E alone (*p*-value cutoff: 10^{-4}). **B**, **C**, **E** Bubble size represents the number of genes in each category, also indicated next to each bubble.

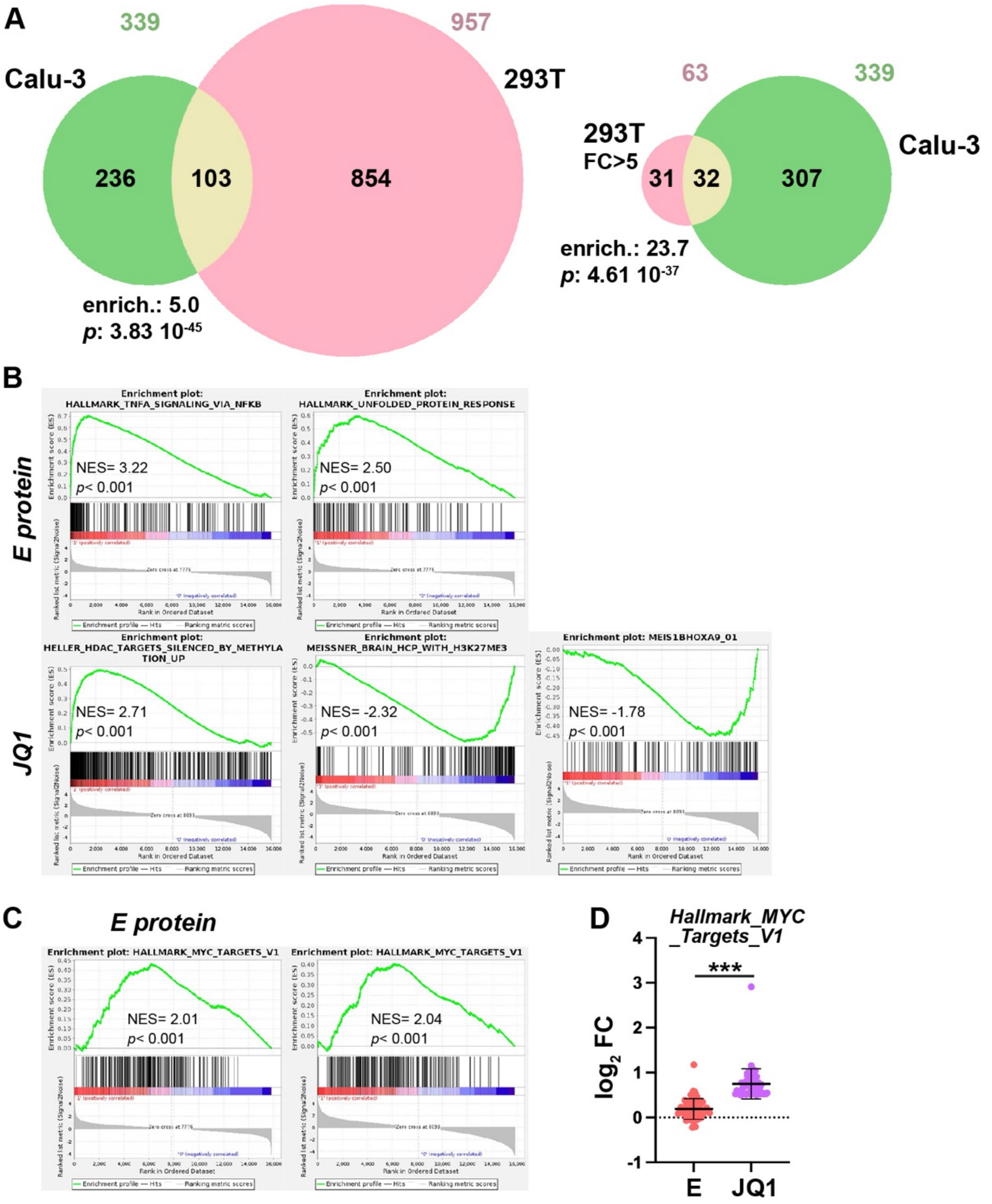

Supplementary Fig. ***S5*** *Gene Set Enrichment Analysis (GSEA) of E overexpression and BET inhibition.* **A** Overlapping of genes upregulated by SARS-CoV-2 infection in Calu-3 cells [24] and genes upregulated (left) or most upregulated (fold change (FC) > 5, right) by expression of E in HEK293T cells, represented by a Venn diagram. Numbers on top of the diagram indicate the total number of upregulated genes in each condition. Enrichment (enrich.) of the overlapping and its associated *p*-value, as determined with the hypergeometric test, are also indicated. **B** GSEA plots from RNA-seq data analysis of misregulated genes after overexpression of E protein or JQ1 treatment. Some significative categories for each condition are shown. **C** GSEA plots of misregulated genes by E overexpression or JQ1 treatment related to Hallmark_MYC_Targets_V1. **B**, **C** Normalized enrichment score (NES) relative to aleatory samples of the same size is shown for the different plots, as well as nominal *p*-value for statistical significance for the enrichment. **D** Representation of $\log_2$ FC values of genes grouping in the category Hallmark_MYC_Targets_V1, upregulated by JQ1 treatment, and compared with values in response to E expression. A mean value ± s.d. for each set of genes under both conditions is shown. Statistical significance was determined by paired Student's *t*-test: *** $p < 0.001$.

Supplementary Table S2 Primers used for RT-qPCR

gene	forward (5’ > 3’)	reverse (5’ > 3’)
ATF3	ATGTCCTCTGCGCTGGAATC	CTTATTTCTTTCTCGTCGCCTCTT
BHLHE40	TGACCGGATTAACGAGTGCAT	TCAATTAGGTTTGTTAGTGCTTTCACA
BST2	GGAGAGATCACTACATTAAACCATAAGCT	ACTTCTTGTCCGCGATTCTCA
HBEGF	CGTGACTTGCAAGAGGCAGAT	GGTGTGGCCAGTGCTTGTG
HEY1	GGAGTGTTGGTGGAAAGGAA	CTCGCACACCATGATCACTT
HSPA5	TGCAGCAGGACATCAAGTTC	ATGTCTTTGTTTGCCCACCT
IFI6	GAGCTGGTCTGCGATCCT	CATCAGGGCACCAATATTACC
ISG15	GAGAGGCAGCGAACTCATCT	GCATCTTCACCGTCAGGTC
MYC	GCTGCTTAGACGCTGGATTT	CCTCGTCGCAGTAGAAATACG
OAS2	CTTAAGAGGCAACTCCGATGGT	ACGTTGGCTTCTCTTCTGATCCT
PPP1R15A	CGCCCAGAAACCCCTACTC	CCAGACAGCCAGGAAATGGA
RPLP0	AACCCTGAAGTGCTTGATATCACA	GCAACAGTTGGGTAGCCAATC
RSAD2	TCCTGCTTGGTGCCTGAATC	ACAGTTCAGAAAGCGCATATATTCAT
SPRY2	TGCACGCCTACAGGTGTGA	TGGGTAGGTGCACTCCTTACATT
